# YAP and endothelin-1 signaling: an emerging alliance in cancer

**DOI:** 10.1186/s13046-021-01827-8

**Published:** 2021-01-09

**Authors:** Piera Tocci, Giovanni Blandino, Anna Bagnato

**Affiliations:** 1 Preclinical Models and New Therapeutic Agents Unit, Advanced Diagnostic and Technological Innovation, Istituto di Ricovero e Cura a Carattere Scientifico (IRCCS), Regina Elena National Cancer Institute, Via Elio Chianesi, 53, 00144 Rome, Italy; 2 Oncogenomic and Epigenetic Unit, IRCCS, Regina Elena National Cancer Institute, Rome, Italy

**Keywords:** Endothelin-1 receptors, β-arrestin1, YAP, Mutant *TP53*, G protein‐coupled receptor

## Abstract

The rational making the G protein-coupled receptors (GPCR) the centerpiece of targeted therapies is fueled by the awareness that GPCR-initiated signaling acts as pivotal driver of the early stages of progression in a broad landscape of human malignancies. The endothelin-1 (ET-1) receptors (ET-1R), known as ET_A_ receptor (ET_A_R) and ET_B_ receptor (ET_B_R) that belong to the GPCR superfamily, affect both cancer initiation and progression in a variety of cancer types. By the cross-talking with multiple signaling pathways mainly through the scaffold protein β-arrestin1 (β-arr1), ET-1R axis cooperates with an array of molecular determinants, including transcription factors and co-factors, strongly affecting tumor cell fate and behavior. In this scenario, recent findings shed light on the interplay between ET-1 and the Hippo pathway. In ET_A_R highly expressing tumors ET-1 axis induces the de-phosphorylation and nuclear accumulation of the Hippo pathway downstream effectors, the paralogous transcriptional cofactors Yes-associated protein (YAP) and Transcriptional coactivator with PDZ-binding motif (TAZ). Recent evidence have discovered that ET-1R/β-arr1 axis instigates a transcriptional interplay involving YAP and mutant p53 proteins, which share a common gene signature and cooperate in a oncogenic signaling network. Mechanistically, YAP and mutp53 are enrolled in nuclear complexes that turn on a highly selective YAP/mutp53-dependent transcriptional response. Notably, ET-1R blockade by the FDA approved dual ET-1 receptor antagonist macitentan interferes with ET-1R/YAP/mutp53 signaling interplay, through the simultaneous suppression of YAP and mutp53 functions, hampering metastasis and therapy resistance. Based on these evidences, we aim to review the recent findings linking the GPCR signaling, as for ET-1R, to YAP/TAZ signaling, underlining the clinical relevance of the blockade of such signaling network in the tumor and microenvironmental contexts. In particular, we debate the clinical implications regarding the use of dual ET-1R antagonists to blunt gain of function activity of mutant p53 proteins and thereby considering them as a potential therapeutic option for mutant p53 cancers. The identification of ET-1R/β-arr1-intertwined and bi-directional signaling pathways as targetable vulnerabilities, may open new therapeutic approaches able to disable the ET-1R-orchestrated YAP/mutp53 signaling network in both tumor and stromal cells and concurrently sensitizes to high-efficacy combined therapeutics.

## Background

G protein-coupled receptors (GPCR) play key roles in different cellular processes. As such, aberrant activation of GPCR can alter the signaling landscape and cellular fate in multiple pathophysiologic contexts including cancer. Consequently, they represent among the membrane receptors the major class of druggable targets [1–3]. The research into GPCR signaling and cancer has been focused to define GPCR protein dynamics through the elucidation of the conformational rearrangements of the GPCR upon receptor stimulation including the status of the intracellular domain and its interacting G-proteins, G-protein coupled receptors kinases (GRKs) and the two β-arrestin isoforms, β-arrestin1 (β-arr1) and β-arrestin2 (β-arr2). Defining the mechanistic dynamics related to GPCR signaling in tumor and microenvironmental contexts is an endeavor for the rational design of high-efficacy cancer therapeutics [4]. Among the GPCR, the endothelin-1 (ET-1) receptors (ET-1R), ET_A_ receptor (ET_A_R) and ET_B_ receptor (ET_B_R), are pervasively expressed in many human malignances and their activation confers to tumor cells peculiar malignant traits, orchestrating the signaling network involved in cell proliferation, cell invasion and migration, drug resistance, angiogenesis and lymphangiogenesis, and metastatization [5, 6]. The binding of 21-amino acid small vasoactive peptides known as endothelins (ETs) activates ET-1R. The ETs group comprises three peptide isoforms endothelin-1 (ET-1), endothelin-2 (ET-2) and endothelin-3 (ET-3) [7–9]. ET_A_R displays more affinity for ET-1 and ET-2, compared to ET-3, while ET_B_R has an equal affinity for the three ETs [7–12]. ET-1 is the predominantly expressed isoform and represents also the most common circulating form of ETs [10]. ET-1 signaling initiation due to the engagement of ET-1R by ET-1 induces ET-1R conformational changes guarantying the ET-1R coupling to their effectors, as G proteins, GRKs, and β-arr1 or β-arr2. In particular, GPCR signaling activation requires several steps that include G-protein activation and GRKs-determined GPCR phosphorylation of serine residues, which leads to β-arr1 or β-arr2 recruitment; thereby preventing G-protein coupling and obstructing G-protein signaling [13–18]. Beside the β-arr1 and 2 canonical roles in directing GPCR desensitization, internalization and G-protein signaling termination, increasing evidence highlights an alternative signaling machinery in which β-arr isoforms act as signal transducers that convert their established protein-protein interaction into signaling pattern independent from G-protein activities. Both β-arr isoforms guide a complex signaling exchange that leads to unique cellular responses providing a selective advantage to tumor cells [15, 17, 19–35]. In spite of the close β-arr structural similarity and the capability to bind a GPCR in a similar way, the two β-arr isoforms do not exhibit entirely overlapping functions in regulating GPCR signaling, due to their different subcellular localization [19, 36–42]. While β-arr1 is localized in both nuclear and cytoplasmic compartment, β-arr2 is excluded from the nucleus, carrying a nuclear export signal (NES) [43]. The diverse β-arr localization profiles affect their interaction pattern, that for β-arr1 includes many transcription factors, co-factors and epigenetic regulators [26–35]. In this landscape, growing evidence discloses how β-arr1 connects the ET_A_R signaling with other pathways fostering ET_A_R-intertwined signalings critically involved in the metastatic progression and drug response in many tumor types, including ovarian cancer [20–38]. β-arr1, acting as an adaptor for different signaling molecules distributed in the cytoplasm and in the nucleus, may delineate the fine-tuned duration and localization of the ET-1-guided pathways, driving the formation of multi-protein complex, that prolongs the ET-1 signaling pattern [5, 35, 44]. Understanding the spatio-temporal control of tumor cell behavior requires information on how signaling complexity is transduced, including the knowledge of the cross-talk with other oncogenic signaling pathways. Indeed, herein we provide an emerging picture in which the paralogous transcriptional cofactor Yes-associated protein (YAP) and transcriptional coactivator with PDZ-binding motif (TAZ), the main downstream effectors of the Hippo pathway, serve as a hub of GPCR signaling orchestrating different oncogenic functions. In particular, we focus on the YAP/TAZ nexus through which ET-1/β-arr1 fosters chemotherapy escape and metastatization. Finally, we outline the potential anti-cancer therapies targeting the ET-1R/YAP/TAZ network and how this therapeutic approach deserves clinical consideration, emphasizing the urgent need to efficaciously targeting ET-1R/β-arr1-engaged signaling pathways related to several hallmarks of cancer. Such analysis may hold the exciting potential to unveil new vulnerabilities facilitating the design of next generation therapies able to dismantle the ET-1R/YAP-generated oncogenic network.

## The ET-1-driven signaling network in tumor cells and tumor‐microenvironment

The ET-1/ET-1R axis hyper-activation endows cells with malignant potential conferring upon them a landscape of features associated with changes in cell fate and acquisition of aggressive traits. ET-1, and other components of the ET-1 axis, is highly expressed in many human malignancies and their expression is associated with tumoral advanced stages [5, 6]. Clinically relevant, in many tumor contexts [5, 33] high levels of ET-1R are associated with a worst prognosis, proving the unfavorable prognostic role of ET-1R. In this framework, it is known that ET-1 axis acts as a survival and pro-proliferative pathway. In particular, in different tumors ET-1, alone or in combination with other tumor-associated growth factors, delivers signals from the cell surface to the nucleus inducing pro-survival transcriptional programs, protecting tumor cells from anti-cancer therapy-induced apoptosis [5, 33, 34, 45–50]. Here, we aim to define how the signaling transduced by ET-1/ET-1R axis stands at the centerpiece of a signaling hub shared by tumor and stromal cells, expanding aspects that are critical for tumor growth and metastatic progression. Such feature may be attributable to the ET-1 axis ability to intercept other signaling pathways, realizing an intricate network that modulates multiple steps related to cancer metastatization [5, 51–53]. Downstream of ET-1R, β-arr1 has now become a reference node able to engage multiple signal transducers from different cell compartments and with various functions, in turn, directing the integration of ET-1R signaling with other pathways. Among the ET-1R/β-arr1-directed functions rank the cell attitude to switch from an epithelial to a mesenchymal phenotype (EMT), that fuels tumor cell migration, invasion and therapy resistance [33, 34, 45, 51–53]. In the array of the ET-1R/β-arr1-integrated signaling, the RTK-activated signaling pathways have been well characterized. Indeed, it has been reported that ET-1/ET1R axis, through the intermediation of β-arr1 that recruits SRC, leads to the transactivation of RTK family members, as the EGFR [20, 21, 41] and the vascular endothelial growth factor-3 (VEGFR-3) [54]. In addition, β-arr1 guides the interplay with different mediators of cytoskeleton dynamic, directing the convergence of ET-1R axis with cytoskeleton remodeling signalings. These integrated pathways produce morphological changes ascribable to the formation and activation of Rho-GTPase-mediated actin-rich invasive protrusions named invadopodia, able to confer an invasive behavior to tumor cells [22–25, 55].

As scaffolding hub, β-arr1 may propagate ET-1/ET-1R signaling in the nucleus, controlling the activity of many transcription factors, as β-catenin [26, 33, 34], or nuclear factor κB (NF-κB) [27]. Similarly, it has been demonstrated that β-arr1, anchoring the hypoxia-inducible factor-1α (HIF-1α), sustains its pro-angiogenic transcriptional schedule [28], portraying the multilayered β-arr1 ability to regulate ET-1-determined transcriptional network, involved in malignant cell behavior.

In addition, mounting evidence suggests that tumor cell behavior is regulated by the dialogue among tumor cells and tumor microenvironmental (TME) elements, and is fueled by the constant interplay between signaling pathways concurrently operating within the tumor. Such signaling networking is becoming a fundamental contributory factor in empowering tumor cells with several hallmarks of cancer. In this perspective, increasing studies shed light on the ability of the ET-1 axis to capture other signaling routes dynamically transducing short communication between tumor cells and neighboring stromal cells [5]. In this regard, recent evidence highlights that the cooperation between ET-1/ET_B_R and VEGF/VEGFR-3 axes affects not only the tumor cells but also the TME. Tumor cells, such as ovarian cancer cells and melanoma cells, release pro-angiogenic factors including ET-1 and VEGF that induce a pro-angiogenic phenotype in endothelial cells (EC) [56–59]. In an autocrine/paracrine fashion, ET-1 via ET_B_R may mediate both early and late angiogenic events. These include blood and lymphatic EC proliferation, invasion and migration and morphogenic changes resembling capillary like-structure tube formation which are necessary for the sprouting of new vessels [56–59]. Additionally, ET-1 signaling may affect both blood and lymphatic EC activities by cooperating with hypoxia stimulus [57, 58]. Indeed, ET-1, similarly to hypoxia and to a greater extent in hypoxic conditions, acts as an angiogenic mediator inducing the hypoxia-inducible factor-1α (HIF-1α) stabilization. The consequent upregulation of the VEGF-C, VEGF-A and VEGFR-3 favors EC growth and differentiation thereby contributing to angiogenesis and lymphangiogenesis [57–59]. More relevant, it has been demonstrated that ET-1R blockade by using macitentan, a dual ET_A_R/ET_B_R receptor antagonist, beside to interfere with ET_A_R-driven tumor growth and progression, is able to impair ET_B_R-mediated vascularization of ovarian cancer xenografts. In particular, macitentan, by inducing the apoptosis of tumor-associated EC, significantly reduces vascular formation *in vivo* [33]. On the other side, it is increasingly clear that ET-1 released from blood and lymphatic EC modulates tumor cell behavior. Indeed, tumor cells exposed to EC conditioned media exhibited an enhanced cell motility and plasticity [59]. Altogether these findings witness the existence of a bilateral interplay between tumor cells and EC in which ET-1 represents one of the soluble components that mediates such communication, favoring the establishment of a permissive environment for tumor cell growth and metastatic progression.

An increasing body of evidences witnesses that the ET-1 system takes part in the reprogramming of the microenvironmental cell behavior affecting cancer-associated fibroblasts (CAF) [60–62] and different subtypes of immune effectors, including competent dendritic cells (DC) [63], tumor-associated macrophages (TAM) [63–65], and tumor-infiltrating lymphocytes (TIL) [66], regulating their maturation and activity [60–65] or interfering with their recruitment to the tumor [66].

For instance, it has been demonstrated that ET-1 released by tumor cells can affect the behavior of CAF that have been isolated from tissues adjacent to different human tumors, such as colon cancer [60], ovarian cancer [61] and breast cancer [62, 63]. In particular, in colorectal cancer it has been shown that ET-1 produced by tumor cells can act on CAF expressing both ET_A_R and ET_B_R, inducing their growth, migration, contraction and production of proteins that modify the architecture of the extracellular matrix (ECM) [60]. In addition, it has been observed that the co-culture of fibroblasts with tumor cells derived from the ascitic fluid of ovarian cancer patients affects fibroblast behavior sustaining their proliferation. Pre-treatment with ET_A_R and ET_B_R receptors antagonists interferes with fibroblast growth, suggesting that such effect is the direct consequence of the inhibition of the paracrine release of ET-1 by tumor cells [61]. Similarly, ET-1 secreted by CAF may influence tumor cell phenotype, as observed in oral cancer, in which CAF-released ET-1 affects the invasive behavior of tumor cells via a paracrine signaling [67].

Interestingly, it has been reported that ET-1 released by ovarian cancer cells may affect the recruitment of TIL to the tumor via ET_B_R, reducing the expression of the endothelial intercellular adhesion molecule 1 (ICAM1), therefore interfering with the TIL homing to the tumor [68]. These observations reveal that ET-1R, mediating the double regulation of the tumor and its surrounding TME, favor tumor development in an accommodating tumor milieu, emphasizing how ET-1 serves as a common communication route between tumor cells and surrounding stromal cells.

## The GPCR/YAP signaling in cancer

GPCR and their related ligands are emerging as critical drivers of the activity of YAP and TAZ, the two main determinants of aggressive traits in tumor cells [69–71], through the downstream G-proteins [71]. The first evidence of GPCR-dependent YAP/TAZ modulation reports that lysophosphatidic acid (LPA) and sphingosine-1-phosphate (SP1) receptors through the associated Gα_12/13_ and Gα_q/11_ may positively regulate YAP/TAZ activity in different human malignancies [69, 72, 73] promoting ovarian cancer cell migration [69] or hepatocellular carcinoma cell proliferation [73]. Starting from these breakthroughs, several GPCR-associated pathways have been shown to regulate YAP/TAZ functions, including G protein-coupled estrogen receptor (GPER) [74] and the protease-activated receptors (PARs) [75, 76]. In the landscape of GPCR bound by secretory proteins, also the angiotensin II receptor AT1 is involved in YAP activation. Indeed, AT1 blockade attenuates tumor cell growth by inhibiting YAP oncogenic activity [77, 78]. The prostaglandin E_2_ receptor (EP2), induces YAP expression and transcriptional activity via the associated Gα_q/11_ promoting cell proliferation in different tumor models [79, 80]. Among the chemokine receptors, the CXCR4/Gα_12/13_ signaling pathway is involved in YAP-mediated EMT [81]. Moreover, in prostate cancer it has been discovered the free fatty acid receptor 1 (FFAR-1)-dependent activation of YAP pathway [82]. Of relevance, the Frizzled receptor upon Wnt binding, activates a Gα_12/13_-driven alternative signaling pathway that leads to LATS1/2 kinases repression and YAP/TAZ activation [83] (Table 1). Despite the emerging importance of the cross-talk between the GPCR and YAP/TAZ signaling pathways in the progression of many human malignancies, the exact mechanisms driving its activation in different contexts remain to be fully resolved.

**Table 1:** The regulation of YAP/TAZ activity by GPCR-associated signaling in human malignancies

GPCRs	Coupling Protein	Cancer Type	Drugs	References
**LPA receptors**	Gα12/13 Gαq/11	Ovarian cancer	Phosphatase-resistant LPA analogues Monoclonal antibodies for LPA	[68, 84–86]
**S1P receptors**	Gα12/13	Ovarian cancer, Hepatocellular carcinoma	Monoclonal antibodies for S1P	[69, 72, 85, 86]
**G protein-coupled estrogen receptor (GPER)**	Gαq/11	Breast cancer	GPER inhibitors (agonist analogous, G15)	[74]
**Protease-activated receptors (PARs)**	Gα12/13 Gαq/11	Breast cancer	RhoA GTPase inhibitors (C3 transferase)	[75, 76]
		Melanoma		
		Colon cancer		
		Lung cancer		
		Pancreatic cancer		
		Prostate cancer		
		Squamous cell carcinoma of the head and neck		
**Angiotensin II Receptor (AT1)**	Gαq/11	Prostate cancer	Angiotensin receptor blockers (losartan)	[77, 78]
		Cholangiocarcinoma		
**Prostaglandin E2 receptor (EP2)**	Gαq/11	Colon cancer	Prostaglandin synthetase blockers (indomethacin)	[79, 80]
		Hepatocellular carcinoma		
		Head and neck cancer		
		Non-small cell lung cancer		
**Chemochine (C-X-C motif) receptor 4 (CXCR4)**	Gα12/13 Gαq/11	Non-small cell lung cancer	CXCR4 antagonists (WZ811)	[81]
		Breast cancer		
		Oral squamous carcinoma		
		Chronic Myelogenous leukemia		
**Free Fatty Acid receptor 1 (FFAR1)**	Gαq/11	Prostate cancer	Docosahexaenoic acid (DHA)	[82]
**Wnt receptor (Frizzled, FRD)**	Gα12/13	Colorectal cancer	RhoA GTPase inhibitors (C3 transferase)	[83]
		Prostate cancer		
		Hepatocellular carcinoma		
**Endothelin A receptor (ETAR)**	Gαq/11	Colon cancer	Selective ETAR antagonists (BQ123)	[87]
**Endothelin A receptor (ETAR)**	β-arr1	Ovarian cancer	Dual ET-1R antagonists (macitentan)	[88, 89]
		Breast cancer		

Notably, growing evidence for YAP and TAZ different functions that are associated with diverging transcriptional programs is emerging [90]. For example, in lung cancer cells YAP is mainly responsible of the transcription of genes involved in tumor cell proliferation, whereas TAZ preferentially modulates genes implicated in ECM organization and cell migration [90], suggesting that YAP and TAZ may direct complementary oncogenic activities.

## The ET-1/YAP network in cancer progression and drug resistance

ET-1/ET-1R axis ranks in the large array of GPCR-generated pathways shown to modulate YAP/TAZ activity. In this regard, it has been reported that ET-1 may induce the expression of well-recognized direct YAP/TAZ common target genes, the connective tissue growth factor (*CTGF*) and cysteine-rich protein 61 *(CYR61*) [91–94]. In particular, it has been demonstrated that ET-1 in primary osteoblasts may induce *CTGF* and *CYR61* transcription promoting osteoblast proliferation and new bone formation [91]. Interestingly, in mouse cardiomyocytes, used as study models for cardiovascular diseases, it has been described that *CTGF* represents an effector of the ET-1-induced fibrosis [92, 93], and in human lung fibroblasts *CTGF* is required for ET-1-induced alpha-smooth muscle actin (α-SMA) expression [94]. These findings suggest that ET-1R may represent an upstream regulatory component of the YAP/TAZ-mediated transcription activation. Consistent with these observations, in colon cancer cells highly expressing ET_A_R, ET-1/ET_A_R-driven YAP/TAZ nuclear accumulation and gene transcription are evidenced. Mechanistically, activated ET_A_R couples the Gα_q/11_ and inhibits LATS1/2 kinases activity, resulting in YAP/TAZ dephosphorylation and activation that favors the tumor growth. These outcomes are impaired by ET_A_R and YAP/TAZ depletions [87]. Interestingly, in uveal melanoma cells ET_B_R activation by ET-3, via a G-protein-transduced signaling, promotes YAP-associated gene transcription [95]. These findings prove the existence of a regulatory network connecting ET-1 system to the YAP/TAZ-driven gene transcription through its association with the DNA-binding TEAD1-4 family members [96]. Recent evidence expands the ET-1R signal transduction repertoire disclosing a sophisticated signaling mediated by β-arr1, in a G-protein independent manner. Indeed, the physical and functional interaction between β-arr1 and YAP downstream of ET_A_R signaling, leads to YAP nuclear accumulation through an alternative route that is not mediated by G-protein. In particular, it has been described how β-arr1 bridges ET-1/ET_A_R axis to YAP signaling in high-grade serous ovarian cancer (HG-SOC) cells and in breast cancer cell lines harboring *TP53* mutations, fostering the YAP/TAZ-dependent transcriptional program that confers upon tumor cells an invasive behavior [88, 97]. In the nuclear compartment β-arr1 enrolls another oncogenic player, mutp53 protein, building up a transcriptional competent complex consisting of β-arr1/YAP/mutp53/TEAD that induces the aberrant expression of target genes, such as *CTGF* and *CYR61*. Notably, in breast cancer cells β-arr1 downstream of ET-1 may also coordinate the interaction of YAP/mutp53 with other active transcriptional factors, such as NFY, regulating its transcriptional repertoire, thus promoting cell proliferation [88, 97]. These evidence indicate that β-arr1-dependent signaling can engender highly characteristic transcriptomic phenotypes and generate long-lasting effects through the formation of multiprotein transcription complexes, composed by β-arr1/YAP/NFY/mutp53 in breast cancer cells, and by β-arr1/YAP/TEAD/mutp53 in HG-SOC cells. It is increasingly clear that β-arr1/YAP/mutp53 complex represents the initial scaffold that integrates and deciphers different stimuli into multiple transcriptional programs, on which transcriptional regulatory networks could be built to dictate different cell fate and behaviors. Thus, the interaction with different transcription factors, tethering mutp53 to the DNA, can expand mutp53 agenda to orchestrate specific gene expression regulating tumor growth and progression. Of relevance, the β-arr1/YAP/mutp53/TEAD target gene pool includes *EDN1*, indeed, the depletion of all the players of such active transcriptional complex, including mutp53, strongly reduces the *EDN1* gene expression, as well as ET-1 promoter activity [88]. The enhanced expression of *EDN1* can promote a self-amplifying vicious cycle potentiating ET-1 axis-dependent adverse outcomes. This observation is supported by previous studies reporting that primary ovarian cancer cells release ET-1 in their conditioned media to a concentration that is within the biologically effective range for this peptide, to ensure the ET-1 binding to the ET-1R. These findings imply that ET-1 sustains tumor growth and progression through an autocrine feed-forward loop that may represent a magnifying persistent mechanism in ovarian cancer cells [98, 99].

Moreover, these findings are in line with recent evidences reporting the existence of the cross-talk between gain of function mutp53 proteins and the Hippo signaling pathway [100]. In particular, it has been described that YAP and mutp53 share a common transcriptional program relevant for sustaining cell proliferation in different human malignancies [93, 101–104]. Therefore, downstream of ET_A_R, β-arr1/YAP/mutp53 complex may be employed to turn on a variety of transcriptional patterns. Clinically relevant, HG-SOC patients carrying *TP53* mutations and simultaneously expressing high levels of ET_A_R, β-arr1 and YAP, face a poor prognosis compared to those patients lacking this molecular signature. These results suggest that such adverse clinical outcomes may be the direct consequence of the integration between ET_A_R/β-arr1 and YAP/mutp53 signaling pathways. Interestingly, downstream of the ET-1 signaling YAP/TAZ has just begun to be recognized as a modulator of the response to anti-cancer therapies. In this regard, it has been described that cisplatinum-resistant ovarian cancer cells acquire platinum resistance through the activation of an adaptive ET_A_R/β-arr1/YAP/TAZ signaling cascade that sustains cell survival, cell plasticity, and lowers cisplatinum sensitivity. Mechanistically, downstream of ET_A_R, β-arr1, instructing the cooperation between the ET-1 axis and RhoA/actin cytoskeleton signaling, guides YAP/TAZ nuclear compartmentalization, favoring a YAP/TAZ/TEAD-committed transcriptional reprogramming that consolidates the treatment evasion [89]. Of clinical interest, the analysis of the integrated ET_A_R (*EDNRA*) and YAP (*YAP1*) gene expression in platinum responder and non-responder ovarian cancer patients, showed that these genes are more expressed in the non-responder than responder patients, suggesting the potential predictive value of this signature [104]. Remarkably, in uveal melanoma cells it has been demonstrated that also the ET_B_R acts as an upstream activator of YAP signaling, representing a therapeutic escape pathway from MEK inhibitors, one of the most explored targeted therapies for uveal melanoma [94].

The control of tumor cell behavior is regulated by a complex network of autonomous and non-cell autonomous signals that converge to establish specific transcriptional programs. Among these signals, YAP and TAZ are able to orchestrate tumor-stroma interactions, instructing specific transcriptional responses. Thus, YAP/TAZ act within tumor cells promoting responses in neighboring stroma, composed of ECM with mechanical features and other cell types, including CAF, endothelial and immune cells [105, 106]. In turn, the activation of YAP/TAZ in different stromal cells creates a corrupted TME that mutually dialogues with tumor cells regulating tumor proliferation, progression and drug response [71]. In particular, it has been recently reported that YAP/TAZ act as immune regulators between immune cells and tumor cells, weakening the tumor immune response, and opening new avenues to regulate tumor immunosuppression [71]. Among the multifaceted roles in the TME, YAP/TAZ not only recruit TAM to the tumor and its surrounding tissue, but regulate its polarization [107]. Tumor cells employ many strategies to evade immune surveillance [108–112]. In particular, it has been demonstrated that YAP/TAZ nuclear translocation in human melanoma [109], breast [110, 111] and lung cancer [111] cells is associated to the transcription of *PDL-1* gene that, in turn, suppresses the T-cell-mediated killing of tumor cells.

**Fig. 1:**
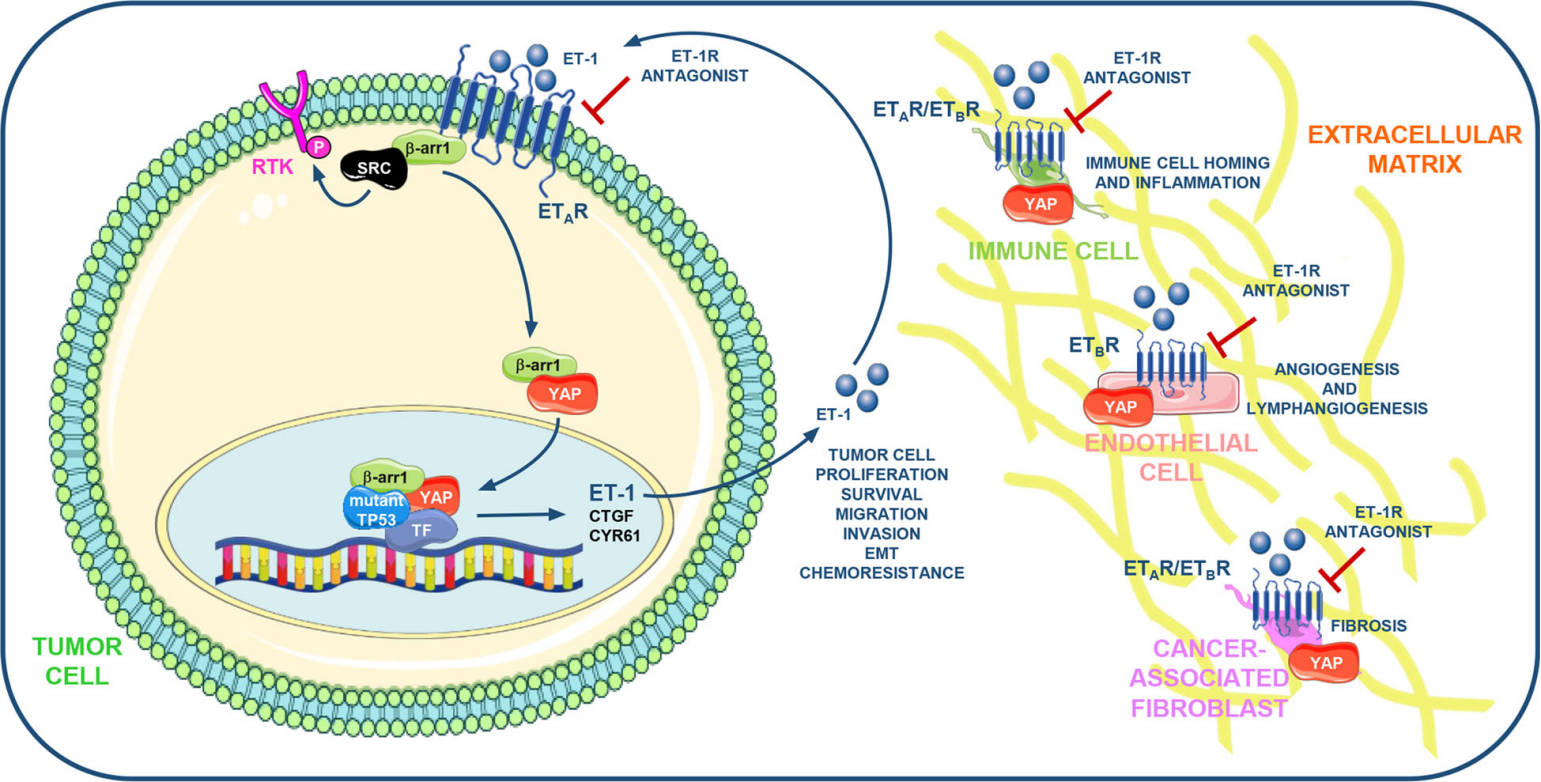
Schematic model illustrating the cross-talk between the ET-1R axis and the YAP pathway. The ET-1R/β-arr1-generated signaling network instructs highly specific transcriptional programs through the binding of specific transcription factors (TF) as TEAD, NFY, and HIF-1α, that impact on the behaviour of cancer cells, promoting the amplification of an ET-1 autocrine vicious circuit, critically involved in tumor cell proliferation, survival, cell invasion and migration, epithelial-to-mesenchymal transition (EMT) and chemoresistance. The autocrine/paracrine release of ET-1 may in parallel impact on tumor microenvironment (TME) elements embedded in the extracellular matrix (ECM), affecting their features. ET-1 activating the ET_B_R expressed by endothelial cells (EC) and lymphatic endothelial cells (LEC) promotes angiogenesis and lymphangiogenesis. Moreover, ET-1R/YAP may sustain the growth, migration and contraction of cancer-associated fibroblasts (CAF), and may favour the maturation and activity of tumor-associated macrophages (TAM), sustaining the production of inflammatory cytokines crucial for tumor metastatization. In addition, ET-1 via ET_B_R may interfere with the recruitment of the tumor-infiltrating lymphocytes (TIL) to the tumor. Moreover, ET-1 sustains the production of proteins that modify the architecture of the ECM. These knowledge render the ET-1R receptors suitable targets for therapeutic interventions based on the use of dual ET-1R antagonists, as macitentan, able to simultaneously interfere with the ET-1R/β-arr1-induced signaling network and YAP in tumor cells and TME elements. Part of the figure is drawn using pictures from Servier Medical Art (https://smart.servier.com), licensed under a Creative Commons Attribution 3.0 Unported License (https://creativecommons.org/licenses/by/3.0)

In this complex scenario, mutual adaptations between tumor cells and their stroma are regulated by the properties of the ECM that affects the cell fate through YAP/TAZ [112]. Hence, YAP/TAZ respond to the physical cues of the ECM as a mechanotransducer decoding a range of inputs not only in tumor cells but also in the TME elements [71], including CAF that promote the induction of ECM stiffening [113–116]. These studies illustrate that tumor cells may employ ECM to deliver mechanosignals that influence YAP/TAZ status in stromal elements and *viceversa* through a persistent circuit, emphasizing how intertwined and sophisticated are the routes that promote YAP/TAZ activation. The ability of soluble factors, as ET-1, to activate YAP could be a direct effect or the indirect result of ECM remodeling. It is renowned that YAP/TAZ can act as sensors of a cell’s physical environment that translates mechanical inputs into gene expression. In this regard, ET-1R is implicated in YAP/TAZ regulation through RhoA GTPases, crucial regulators of the assembly dynamics and functions of the actin cytoskeleton [22–25, 89]. Moreover, recent evidence suggests that GPCR can sense mechanical cues, as the pressure, and participate in mechanical force regulation of YAP/TAZ activation [117]. These findings allow us to hypothesize that ET-1R may also initiate a mechanosignals flow that via ECM guides YAP/TAZ activation in both tumoral and stromal compartments. Better defining of the GPCR regulation in both tumor and stromal cells, through the cross-talk with YAP and TAZ that orchestrate the bi-directional tumor-stromal cell interactions, might improve the characterization of targetable vulnerabilities. Although, currently only few data are available to document the adverse consequences generated by the integration between the ET-1R axis and the YAP and TAZ signaling in the tumor ecosystem (Fig. 1), the preclinical findings [87–89, 95, 97] raise the possibility that ET-1R blockade could be effective against ET-1R/YAP/TAZ-driven tumors, ameliorating the outcomes of the patients.

## Targeting GPCR/YAP signaling axis for anti‐cancer therapy

Given that GPCR and YAP/TAZ signaling are frequently dysregulated in cancers and considering the discovered partnership between these pathways in cancer progression and therapy resistance, the repurposing of currently available GPCR-based drugs may embody a promising anti-cancer therapeutic strategy blunting YAP/TAZ-driven activity, a goal still far from being achieved. In this scenario, many therapeutic approaches have been developed to interfere with the GPCR/YAP/TAZ signaling network. A potential therapeutic route is represented by the use of drugs able to target proteins involved in the transduction of the GPCR signaling. For instance, it has been observed that the antagonization or depletion of Gα_12/13_- or Gα_q/11_-mediated signaling by using phosphatase-resistant LPA analogues or antibodies specific for LPA or SP1 receptors may interfere with YAP/TAZ activation [85, 86]. Recently, it has been reported that a cyclic depsipeptide, FR900359, targeting Gα_q/11_, interferes with YAP nuclear functions stimulating its inhibitory phosphorylation [118, 119]. An alternative therapeutic solution to hinder YAP/TAZ may be represented by the activation of another effector of GPCR signaling, the protein kinase A. Mechanistically, the enhancement of cyclic AMP (cAMP) levels, by using the cAMP activator forskolin, induces PKA activation and LATS kinases functions, inhibiting YAP/TAZ activity [86]. Rho GTPases are now recognized as critical players of GPCR signaling-dependent YAP/TAZ pathway regulation, and statins, known inhibitors of HMG-CoA reductase, inactivating Rho GTPases, may indirectly interfere with YAP/TAZ nuclear accumulation [86, 120, 121]. In this review, we have highlighted the emerging roles of YAP/TAZ as critical effectors of ET-1/ET-1R signaling shared by tumor and stromal cells. In particular, the signaling cross-talk between ET_A_R/β-arr1 axis and the YAP/TAZ pathway generates a YAP/TAZ-dependent highly specific transcriptional program that sustains the invasive behavior and the drug tolerant state of OC cells [88, 89]. The identification of such YAP/TAZ-activating signaling suggests that the targeting of ET-1R/β-arr1-associated signaling may represent an attractive therapeutic option able to inhibit YAP/TAZ activation. Promising studies in different preclinical tumor models featured the benefit of the dual ET_A_R/ET_B_R antagonists. Among these, macitentan, FDA approved for the pulmonary arterial hypertension (PAH), is able to engender chemosensitivity and responsiveness to target agents in different preclinical settings [5, 122–129]. In patient-derived (PD) HG-SOC cells, harboring hot spot *TP53* missense mutations and in PD xenografts, macitentan, preventing the β-arr1-orchestrated signaling network, hampers YAP/TAZ cytoplasmic-nuclear shuttling, disrupts YAP/mutp53 transcriptional activity, inhibits metastatic spread and re-sensitizes chemoresistant HG-SOC cells to platinum-based therapy [88, 89]. Of note such effects are due to macitentan ability to dismantle YAP and mutp53 oncogenic network [88, 89] (Table 1). Considering that *TP53* is frequently mutated in many human malignancies, the use of dual ET-1R antagonists holds the potential for the treatment of *TP53* mutant cancer patients.

Taking into account that YAP and TAZ can orchestrate non overlapping transcriptional programs regulating cellular outcomes [90], future investigations are needed to uncover the different activities of YAP and TAZ downstream of GPCR, as ET-1R, including the oncogenic network with mutp53. Together these findings suggest that breaking this oncogenic cross-talk driven by ET-1/ET-1R axis might be exploited in rational and tolerable combination treatment strategies in the clinical setting.

## Conclusions

An intriguing scenario depicts the cross-talk between ET-1 signaling and YAP/TAZ that influences tumor cell behavior and signaling interactions with microenvironmental neighboring cells controlling fine-tuned mutual regulation of cell fate decisions. The ability of YAP/TAZ to directly control the transcription of ET-1R ligand, contributes to understand how the β-arr1-mediated signals control the complex spatio-temporal regulation of tumor cell plasticity. Although how ET-1R/β-arr1 and YAP/TAZ signaling are integrated within different contexts is still not well-defined, we can envision that this interplay occurs between distinct cell types, as stromal and epithelial cells embedded in the ECM, adding complexity to the emerging model (Fig. 1). Taking into consideration that most of the results obtained are not in living tissues, new approaches as *in situ* single-cell RNA sequencing will be critical to further dissect the interplay of ET-1/YAP/TAZ in context-dependent tumor. Stromal cells, immune cells and the ECM act in concert to establish a niche that facilitates tumor cell plasticity. Therefore, to examine the influence of TME on tumor cell fate, novel 3D models, as organoids co-cultured with TME elements, may provide a unique platform to study the contribution of non-epithelial components to tumor cell behavior, with the potential to value therapy response [130, 131]. Finally, the discovery of YAP/TAZ as central hub of integrated signaling networks interconnected within the TME suggests that the identification of its drivers may lead to the discovery of new therapeutic targets [71]. To date there are few active clinical trials that evaluate drug repurposing able to inhibit YAP/TAZ activity. This prospectively supports that ET-1R-directed therapeutic intervention can be an exciting challenge to disable YAP/TAZ-driven oncogenic transcription; thereby preventing metastasis formation and acquisition of drug resistance. Consequently, ET-1R targeting by the dual ET-1R antagonists might have immediate therapeutic implications blunting YAP/mutp53 oncogenic activities in diverse human cancers.

## Data Availability

Not applicable.

## Abbreviations

α-SMA: Alpha-smooth muscle actin
β-arr1: β-arrestin1
β-arr2: β-arrestin2
CAF: Cancer associated fibroblasts
cAMP: Cyclic AMP
CYR61: Cysteine-rich protein 61
CTGF: Connective tissue growth factor
DC: Dendritic cells
EDN1: ET-1 gene
ICAM1: Endothelial intercellular adhesion molecule 1
EC: Endothelial cells
ET: Endothelins
ET-1: Endothelin-1
ET-2: Endothelin-2
ET-3: Endothelin-3
ET-1R: Endothelin receptors
ET_A_R: Endothelin A receptor
ET_B_R: Endothelin B receptor
EGFR: Epithelial growth factor receptor
EMT: Epithelial-to-mesenchymal transition
ECM: Extracellular matrix
FFAR-1: Fatty acid receptor
GPCR: G protein-coupled receptors
GPER: G protein-coupled estrogen receptor
GRKs: G protein-coupled receptors kinases
HG-SOC: High-grade serous ovarian cancer
HIF-1α: Hypoxia-inducible factor-1 α
HMGCR: HMG-CoA reductase
LEC: Lymphatic endothelial cells
LPA: Lysophosphatidic acid receptor
MAPK: Mitogen-activated protein kinase
mutp53: Mutant p53
NSCLS: Non-small cell lung cancer
NES: Nuclear export signal
PD: Patient derived
PI3K: Phosphoinositide 3-kinase
EP2: Prostaglandin E_2_ receptor
PARs: Protease-activated receptors
PKA: Protein kinase A
AKT: Protein kinase B
PAH: Pulmonary arterial hypertension
SP1: Sphingosine-1-phosphate receptor
TAZ: Transcriptional coactivator with PDZ-binding motif
TME: Tumor microenvironment
TAM: Tumor-associated macrophages
TIL: Tumor-infiltrating lymphocytes
RTK: Tyrosine kinase receptors
VEGF: Vascular endothelial growth factor
VEGFR-3: Vascular endothelial growth factor receptor
YAP: Yes-associated protein
